# Management of giant pseudomeningoceles after spinal surgery

**DOI:** 10.1186/1471-2474-11-53

**Published:** 2010-03-21

**Authors:** Yi-Jan Weng, Chin-Chang Cheng, Yen-Yao Li, Tsung-Jen Huang, Robert Wen-Wei Hsu

**Affiliations:** 1 Department of Orthopedic Surgery, Chang Gung Memorial Hospital at Chiayi, Taiwan, No 6, West, Chiapu Road, Putz, Chiayi (613), Taiwan; 2 College of Medicine, Chang Gung University, Taoyuan, Taiwan, No 259 Wenhwa 1st Road, Kweishan, Taoyuan (333), Taiwan; 3 Department of Orthopedic Surgery, Xiamen Chang Gung Hospital, Fujian, China, No 123, Xiafei Road, Xinyang Industrial Zone, Haicang District, Xiamen, Fujian (361026), China

## Abstract

**Background:**

Pseudomeningoceles are a rare complication after spinal surgery, and studies on these complex formations are few.

**Methods:**

Between October 2000 and March 2008, 11 patients who developed symptomatic pseudomeningoceles after spinal surgery were recruited. In this retrospective study, we reported our experiences in the management of these complex, symptomatic pseudomeningoceles after spinal surgery. A giant pseudomeningocele was defined as a pseudomeningocele >8 cm in length. We also evaluated the risk factors for the formation of giant pseudomeningoceles.

**Results:**

All patients were treated successfully with a combined treatment protocol of open revision surgery for extirpation of the pseudomeningoceles, repair of dural tears, and implantation of a subarachnoid catheter for drainage. Surgery-related complications were not observed. Recurrence of pseudomeningocele was not observed for any patient at a mean follow-up of 16.5 months. This result was confirmed by magnetic resonance imaging.

**Conclusions:**

We conclude that a combined treatment protocol involving open revision surgery for extirpation of pseudomeningoceles, repair of dural tears, and implantation of a subarachnoid catheter for drainage is safe and effective to treat giant pseudomeningoceles.

## Background

Pseudomeningocele is an uncommon complication of spinal surgery [1-3]. It is an extradural accumulation of cerebrospinal fluid (CSF) in the soft tissue of the back that extravasates through the dural tear [4,5]. Three types of pseudomeningocele (congenital, postoperative and traumatic pseudomeningoceles) have been reported [2,6]. Pseudomeningoceles often occur as a complication of lumbar spinal surgery [7]. The post-laminectomy pseudomeningocele was first described in 1946 by Hyndman and Gerber in a study of extradural cysts [8]. The exact incidence of postoperative pseudomeningocele is unknown because many of these patients are asymptomatic [7]. Another more likely reason is that spine surgeons are reluctant to publish negative results. Pseudomeningocele can present with headache, and sometimes nausea and vomiting [5]. A trapped nerve root of the pseudomeningocele can also occur and cause radicular pain [9].

Giant pseudomeningoceles have rarely been reported and are not well discussed [10]. Optimal treatment of pseudomeningoceles remains controversial. Surgical procedures for treating pseudomeningoceles have been described [2,7,11-13]. In general, pseudomeningoceles are surgically explored and entered. Nerve roots are gently dissected free then reduced into the thecal sac if herniated into the pseudomeningocele cavity. Identification and removal of fistulous tracts is carried out. The dural tear is repaired with a primary suture if the tear is simple and easy to repair, or repaired using a patch of deep fascia if very large or located laterally. Interrupted figure-of-eight sutures of the myofascial layer are used to provide a watertight closure. A subarachnoid catheter is passed through skin, muscle and fascia to lie over the previous dural tear for drainage [2,7,11-13].

**Figure 1:**
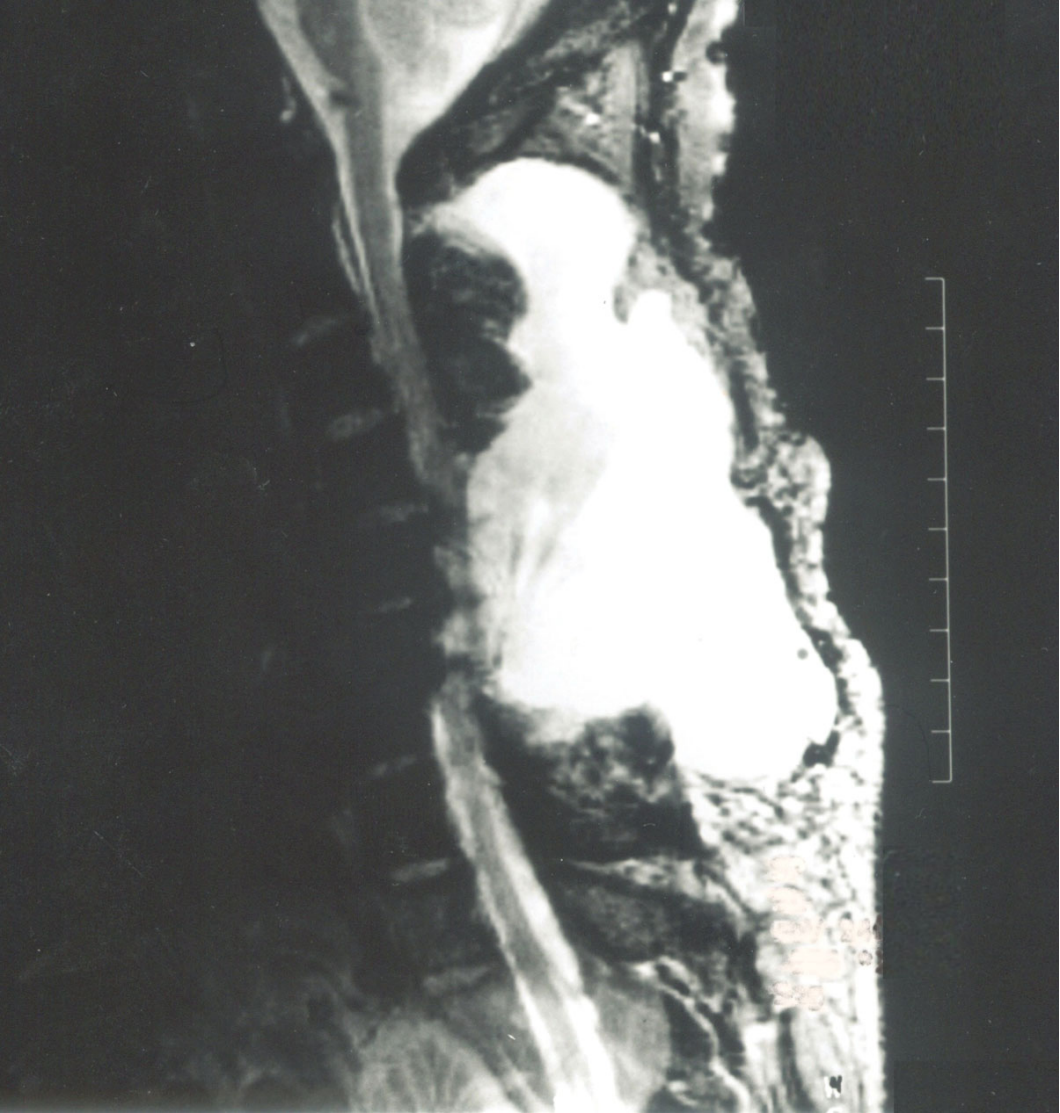
**A giant pseudomeningocele noted after surgery for C5-C6 recurrent disk herniation**. A 53-year-old male underwent cervical laminectomy and discectomy for C5--C6 recurrent disk herniation. After surgery, a giant cervical pseudomeningocele (length, 11 cm; width, 5 cm; depth, 5 cm) was noted on T2-weighted MRI.

After analysis of symptomatic patients with pseudomeningoceles, we defined pseudomeningoceles >8 cm in length as a giant pseudomeningoceles (Figure 1). These giant pseudomeningoceles were managed with a combined treatment protocol: extirpation of the pseudomeningocele, repair of dural tears, and an indwelling a subarachnoid catheter for drainage. The purpose of the present study was to evaluate the clinical results of treating giant pseudomeningoceles by the surgical method descried above. Risk factors for the formation of giant pseudomeningocele were also discussed.

## Methods

Between October 2000 and March 2008, we recruited and evaluated 11 patients with symptomatic pseudomeningoceles after various spinal surgeries. A combined treatment of open revision surgery for extirpation of the pseudomeningocele, repair of dural tears, and implantation of a subarachnoid catheter for drainage was carried out for all patients. This study was approved by the Institutional Review Board of the Chang Gung Memorial Hospital (study reference number, 98-1916B). Written informed consent was obtained from all patients enrolled in the study.

**Figure 2:**
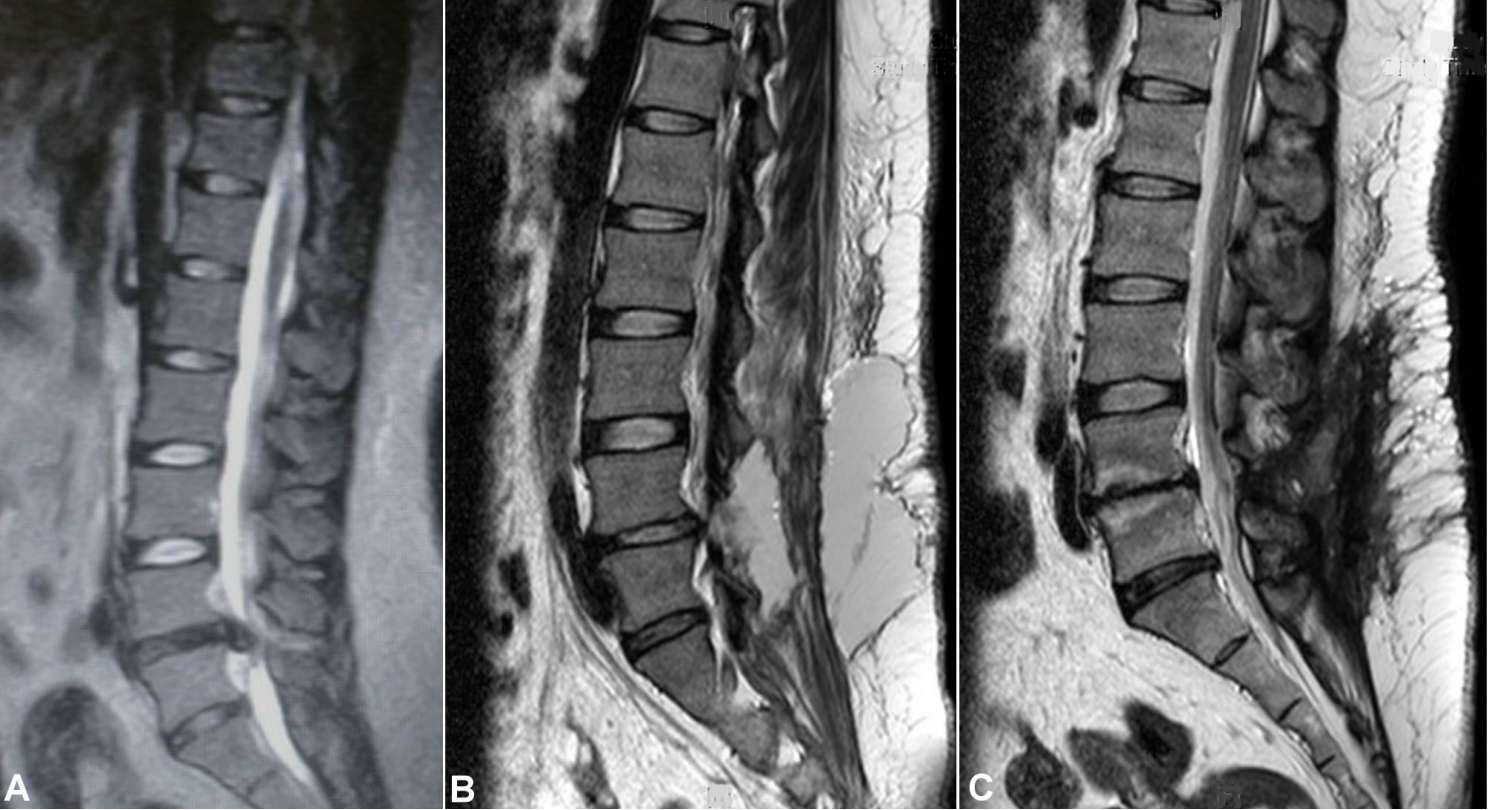
**A giant pseudomeningocele noted after L4-L5 discectomy**. (A) A 26-year-old obese female, body height of 170 cm, body weight 110 kg, and a BMI of 38.1 kg/m^2^. She underwent L4--L5 laminotomy and discectomy for lumbar disk herniation. (B) She developed a postoperative giant lumbar pseudomeningocele (length, 9 cm; width, 8 cm; depth, 7 cm). (C) By extirpation of the cyst, dural repair and subarachnoid drainage, the pseudomeningocele was successfully treated and confirmed on MRI at 3-month follow-up.

Patients were determined to have a pseudomeningocele if there was clinical evidence of a bulging mass with a ballotable collection of fluid. Signs and symptoms were also recorded. Preoperative magnetic resonance imaging (MRI) was done to confirm a fluid collection connecting with the dural sac (Figure 2B). This was repeated 3 months' later (Figure 2C) to evaluate the efficacy of surgery.

In this retrospective study, we collected the demographic and preoperative characteristics (Table 1) and clinical characteristics (Table 2) of patients, as well as variables related to postoperative pseudomeningoceles (Table 3). Outcomes after the combined treatment protocols were evaluated.

**Table 1:** Demographic data and preoperative characteristics in 11 patients with giant pseudomeningoceles

Characteristics	N = 11
**Sex**: N (%)	
Male	7 (63.6%)
Female	4 (37.4%)
	**Mean ± SD (range)**
**Age **(years)	40.8 ± 17.0 (19-68)
**Body Height **(cm)	166.1 ± 10.4 (147-180)
**Body Weight **(kg)	76.3 ± 15.7 (55-110)
**BMI **(kg/m^2^)	27.6 ± 4.9 (21.0-38.1)
BMI > 24.0	9 (82%)

N = number of patients, SD = standard deviation,

BMI = body mass index

**Table 2:** Perioperative data of the initial surgery carried out in 11 patients with giant pseudomeningoceles

Characteristics	N = 11
**Indication of Initial Operation**: N (%)	
HIVD	8 (73%)
Recurrent HIVD	1 (9%)
Spondylolisthesis with Spinal Stenosis	2 (18%)
**Level of Lesion**: N (%)	
Cervical Spine	2 (18%)
Lumbar Spine	9 (82%)
**Initial Operation**: N (%)	
Laminotomy/Laminectomy + Discectomy	9 (82%)
Laminectomy + P-L fusion + TPS Fixation	2 (18%).
**Dural Tear**: N (%)	11 (100%)

N = number of patients

HIVD = herniated intervertebral disc

P-L fusion = posterolateral fusion

TPS = transpedicular screw.

**Table 3:** Perioperative data of 11 patients with giant pseudomeningoceles

Parameters	N = 11
**Signs**: N (%)	
Bulging Mass	11 (100%)
**Symptoms**: N (%)	
Neck Pain/Back pain	7 (64%)
Headache	6 (55%)
Nausea/Vomiting	4 (36%)
Limb Pain/Numbness	2 (18%)
None of symptom	Nil
**Time between signs/symptoms and first surgery (days)**	50.6 ± 20.5 (19-99)*
**Sizes of Pseudomeningocele**	
Length (cm)	8.9 ± 1.2 (8-11)*
Width (cm)	5.7 ± 1.1 (5-8)*
Depth (cm)	4.3 ± 1.3 (3-7)*
**Dural repair with patch deep fascia**	3 (27%)
**Culture of Pseudomeningocele (ORSA)**: N (%)	1 (9%)
**Complications**	
Neurological Deficit	Nil
Wound Infection/Deep infection	Nil
**Follow-up **(mo)	16.5 ± 8.8 (6-30)*
**Recurrence**	Nil

N = number of patients, SD = standard deviation, *: Mean ± SD (range)

CSF = cerebrospinal fluid

ORSA = oxacillin-resistant *Staphylococcus aureus*

## Results

Table 1 lists the demographic data and preoperative characteristics of the 11 (7 males and 4 females) patients in the study group. The mean age of the patients was 40.8 years (range, 19-68 years). Mean body weight and mean height were 76.3 kg and 166.1 cm, respectively. The mean body mass index (BMI) was 27.6 kg/m^2 ^(range, 21.0-38.1 kg/m^2^).

Table 2 details perioperative data of the final surgery before pseudomeningocele formation before the present study was carried out. Indications for the final surgery in 11 patients were herniated intervertebral disc (HIVD) (8 patients; 73%), recurrent HIVD (1; 9%), and spondylolisthesis with spinal stenosis (2; 18%). The levels of lesions indicated for surgery were cervical spine (2; 18%) and lumbar spine (9; 82%). The type of surgery carried out for these patients was laminotomy/laminectomy and discectomy (9; 82%), and laminectomy and posterolateral fusion with transpedicular screw fixation (2; 18%). The number of surgical procedures before pseudomeningocele formation was once 91% of cases and twice in the remaining 9% of cases. Dural tears during the final surgery were noted in all the patients who subsequently sustained postoperative pseudomeningoceles (although the entire dural tear had been primarily repaired at that time).

**Figure 3:**
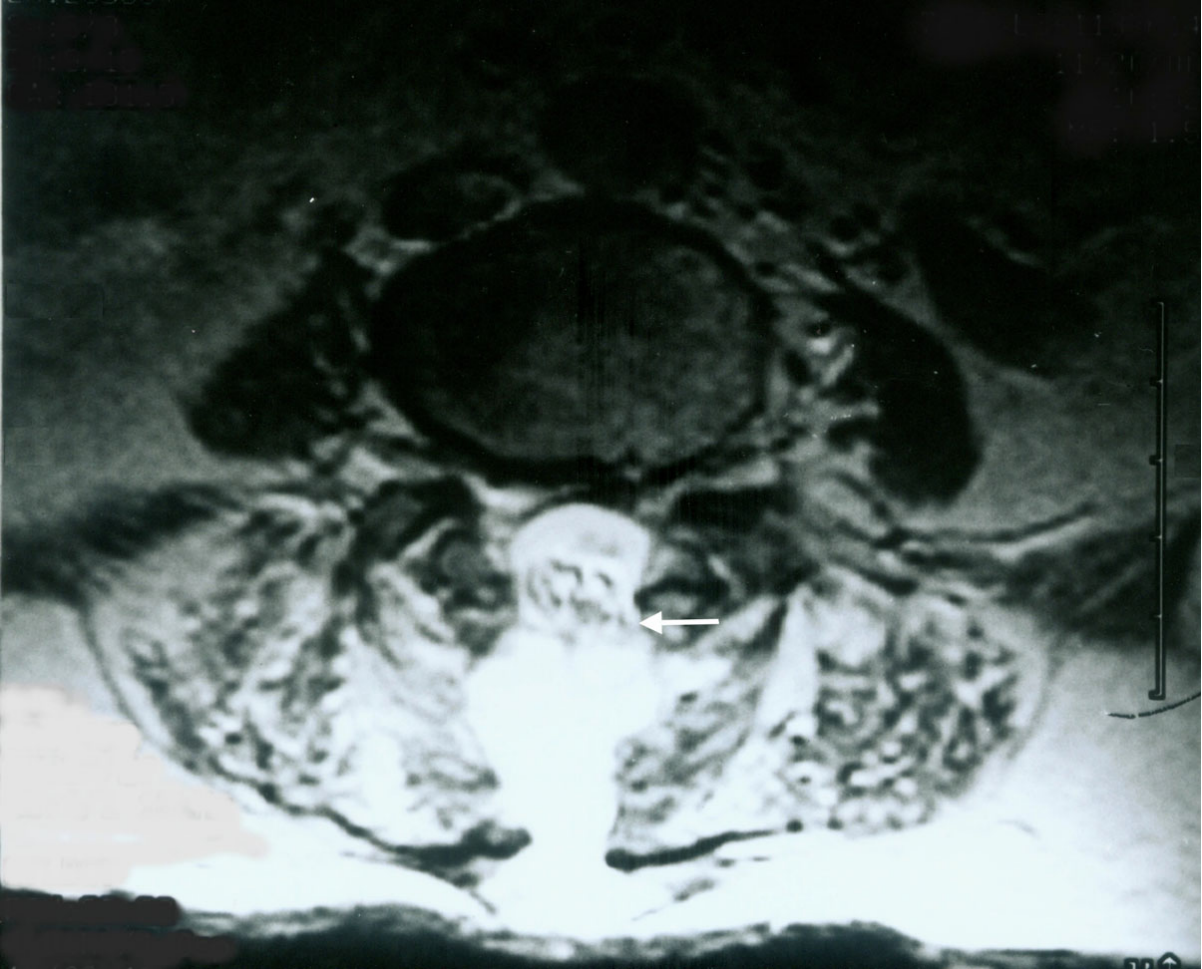
**Nerve roots herniating into a pseudomeningocele**. A 78-year-old male underwent laminectomy and discectomy for L4--L5 disk herniation. A giant pseudomeningocele (length, 9 cm; width, 5 cm; depth, 3 cm) developed after surgery. Nerve roots herniating through the dural and arachnoid tears and into the pseudocyst was observed on axial MRI (arrow).

Analyses of the perioperative data of patients in whom pseudomeningoceles occurred after spinal surgery are presented in Table 3. A bulging mass was noted in all patients, and confirmed as a giant pseudomeningocele by MRI carried out before the combined treatment protocol. The commonest symptoms were pain in the neck or back (63%), headache (55%), nausea or vomiting (36%) and pain or numbness in the limbs (18%). One patient with pain and numbness in the legs who underwent MRI was shown to have a trapped nerve root within the pseudomeningocele (Figure 3). The mean sizes of the pseudomeningoceles were 8.9 cm (range, 8-11 cm) in length, 5.7 cm (range, 5-8 cm) in width, and 4.3 cm (range, 3-7 cm) in depth as measured by MRI (Figure 1). In the current revision surgeries for giant pseudomeningoceles, for 3 patients (27%) with complex and/or laterally located dural tears, a patch of deep fascia graft was used to repair the tear. There was one culture-confirmed infection as oxacillin-resistant *Staphylococcus aureus *in one patient with pseudomeningocele. Complications such as neurological deficit, wound infection, or deep infection in patients receiving the combined treatment protocol after a mean follow-up of 16.5 months was not observed. A recurrence of pseudomeningocele during the follow-up period was not observed for this study population.

## Discussion

Postoperative pseudomeningocele was first reported by Hyndman and Gerber in 1946 in a survey of extradural cysts [8]. They classified extradural pseudocysts into two types: iatrogenic and traumatic. The terms "meningocele spurious", "pseudocyst" or "false cyst", have also been used to describe pseudomeningoceles [2,6,14]. Three types of pseudomeningocele (congenital, iatrogenic and traumatic) were reported by Miller et al. in 1968 [2]. Most pseudomeningoceles are usually associated with neurofibromatosis and Marfan syndrome, and are congenital in origin [1,3,8,15,16]. Congenital pseudomeningoceles tend to occur in the thoracic or thoracolumbar area [1]. An iatrogenic pseudomeningocele typically occurs as a complication after laminectomy of the lumbar spine [7]. Traumatic pseudomeningoceles are rarest, and are more common in the cervical region (but can also occur in the lumbosacral region after a severe distraction injury) [17,18]. The incidence of pseudomeningocele after laminectomy ranges from 0.068% (as reported by Swanson et al. in a series of 1,700 laminectomy patients [19]) to 2% (as reported by Teplick et al. in a series of 400 symptomatic post-laminectomy patients [20]).

Postoperative pseudomeningoceles may result from a tear in the dural mater and pia arachnoid that is unnoticed and is left open during surgery [20-23]. If the dural mater and pia arachnoid are torn, CSF extravasates into the paraspinal soft-tissue space. The CSF may be absorbed initially but, after progressive reactions in the connective tissue of the surrounding tissue, CSF is absorbed less readily, resulting in pseudomeningocele formation [2,5,14,20]. More cases of symptomatic, giant pseudomeningoceles developed in the lumbar region (9 out of 11) than other areas (Table 2). This observation is in accordance with other studies [3,7,9,24]. This may be because CSF in the lumbar region is under a higher hydrostatic pressure than that in the cervical spine in the upright posture, and because more surgical procedures are carried out on the lumbar spine [25].

Patients with pseudomeningoceles may present a wide variety of signs and symptoms (though most patients are asymptomatic). Some pseudomeningoceles can be diagnosed based on the fluctuant mass that enlarges with the Valsalva maneuver (e.g., coughing or sneezing) [26]. The common symptoms of postoperative pseudomeningocele are postural headache, localized back pain and radiculopathy. Nerve roots may subsequently herniate through the dural and arachnoid tears, strangulation of the nerve roots within the cyst may lead to radicular pain and motor deficits [12,23]. Headaches, which are frequently a symptom of large pseudomeningoceles [5], may be the result of a reduction in CSF volume and lowered intracranial pressure [27]. Entrapment of nerve roots in the pseudomeningocele (Figure 3) and pseudomeningocele size seem to be the major factors that determine whether the pseudomeningocele causes symptoms. All of the 11 patients with a giant pseudomeningocele had signs and symptoms (Table 3).

A pseudomeningocele should be considered for patients with recurrent back pain, radicular pain, or a persistent headache after spinal surgery. Most authors consider MRI to be the most effective non-invasive diagnostic tool that can accurately assess the size and location of pseudomeningoceles. MRI was carried out before the combined treatment protocol and was repeated 3 months after surgery to confirm the formation, resolution and recurrence of giant pseudomeningoceles (Figure 2).

The treatment of pseudomeningocele is controversial, particularly in asymptomatic patients. Optimal management of a pseudomeningocele is dependent upon many factors, including sac size, location and symptoms [18]. Small pseudomeningoceles associated with minimal symptoms has been reported to require no treatment [7,28]. The option of conservative management in asymptomatic patients with a pseudomeningocele was accepted because even large pseudomeningoceles can be seen to "scar down" and resolve over time. Non-surgical management should be the preferred approach if the patient is asymptomatic. Early symptomatic pseudomeningoceles associated with a CSF fistula can be treated with spinal drainage [11]. Symptomatic pseudomeningoceles weeks-to-months after initial surgeries may be treated with surgical dural repair [7,11,20]. Aoki reported the treatment of ten patients with postoperative pseudomeningocele by a lumbar shunt [13]. Percutaneous subarachnoid drainage has also been successfully employed to repair dural cutaneous fistulas and early pseudomeningoceles [5]. This procedure can help to create a seal at the leakage site and promote healing by CSF diversion. Primary closure of large dural defects (particularly lateral defects) is difficult. Most authors agree that large dural defects may be closed with patch techniques using autologous tissue, dural allografts, or fibrin glue along the suture line [5,12,29]. Removal of pseudomeningoceles and the release of cord or root is required if they adhere to dura.

There are few reports of giant pseudomeningoceles [10]. We reported a combined treatment protocol of open revision surgery for extirpation of pseudomeningoceles, repair of dural tears, and implantation of a subarachnoid catheter for drainage to treat giant pseudomeningoceles. The combined treatment was safe and effective. Surgical complications and recurrence of pseudomeningoceles were not observed at a mean follow-up of 16.5 months (Table 3). Evidence of signs and symptoms indicating a recurrence of pseudomeningocele in these patients was also not observed.

Analyses of the BMI of these patients revealed that being overweight may be a risk factor for the formation of giant pseudomeningoceles after spinal surgery. The mean BMI of the 11 patients was 27.6 kg/m^2 ^(range, 21.0-38.1 kg/m^2^) (Table 1). For 9 out of the 11 (82%) patients, the BMI was >24.0. Large dural tears with persistent CSF leak, extensive surgical exposure with spinal implants, and revision spinal surgery may also contribute to the formation of giant pseudomeningoceles. Dural tears were identified in all 11 patients at the time of initial surgery that led to subsequent symptomatic pseudomeningocele formation and re-operation. This implied that the initial leaks of CSF were not completely closed even though the entire dural tear had been repaired by primary closure at that time. This emphasizes the importance of adequately training spine surgeons to appropriately address intraoperative CSF leaks. A careful surgical approach is important during revision spinal surgery. Dural tears should be closed first; implantation of a subarachnoid catheter for drainage and repair of soft tissue must be done carefully to prevent a large collection of CSF and constriction of the cauda equina.

Delayed infection of pseudomeningocele was reported by Koo et al. in 1989 [30] and James et al. in 1996 [15]. One of the 11 patients in the present study suffered from CSF infection after the second surgery. This resolved after combining intravenous antibiotics, dural repair with a patch of deep fascia, and placement of a lumbar subarachnoid drain.

Some limitations of the present study must be acknowledged. Comparisons among different treatments such as conservative management, epidural blood patch, subarachnoid drainage alone, and surgery for giant pseudomeningoceles should be obtained. Indications of open surgery dependent upon pseudomeningocele size should also be carried out. Long-term follow-up studies are warranted to determine if the combined treatment protocol is an effective and safe procedure.

## Conclusions

A combined treatment protocol involving open revision surgery for extirpation of the pseudomeningocele, repair of dural tears, and implantation of a subarachnoid catheter for drainage is safe and effective to treat giant pseudomeningoceles.

## Competing interests

The authors declare that they have no competing interests.

## Authors' contributions

Y-JW conceived the study and drafted the manuscript. C-CC and Y-YL participated in the design of the study, assisted in the surgery, and analyzed the radiographic measurements. T-JH participated in the design of the study, carried out surgeries, and approved the final version of the manuscript. RW-WH participated in the design of the study and coordinated the research groups. All authors read and approved the final manuscript.

## Pre-publication history

The pre-publication history for this paper can be accessed here:

http://www.biomedcentral.com/1471-2474/11/53/prepub
